# CD19 CAR-T Cells With Membrane-Bound IL-15 for B-Cell Acute Lymphoblastic Leukemia After Failure of CD19 and CD22 CAR-T Cells: Case Report

**DOI:** 10.3389/fimmu.2021.728962

**Published:** 2021-10-07

**Authors:** Yao Sun, Yongfeng Su, Yizhi Wang, Na Liu, Yuhang Li, Jianlin Chen, Zhuoqing Qiao, Jingwen Niu, Jiangwei Hu, Bin Zhang, Hongmei Ning, Liangding Hu

**Affiliations:** ^1^ Department of Hematology, The Fifth Medical Center of Chinese People's Liberation Army (PLA) General Hospital, Beijing, China; ^2^ Institute of Hematology, The Fifth Medical Center of Chinese People's Liberation Army General Hospital, Beijing, China

**Keywords:** chimeric antigen receptor-T cells, IL-15, B-ALL, extramedullary relapse, CD19, CD22

## Abstract

**Objectives:**

At present, reinfusions of chimeric antigen receptor (CAR)-T cell have exhibited limited efficacy, while their efficacy on extramedullary relapse remains to be further elucidated in B-cell acute lymphoblastic leukemia (B-ALL). Although combination with IL-15 demonstrated the potential to enhance antitumor activity of CAR-T, the efficacy of this approach remains to be validated clinically.

**Methods:**

We reported a patient with B-ALL with extramedullary relapse after allogeneic stem cell transplantation and who was resistant to chemotherapy and radiotherapy. In total, he received four treatments with CAR-T cells repeatedly under the status of disease progression.

**Results:**

First, the patient received autologous murine CAR19-CD28-CD3ζ-T cells and achieved full resolution of extramedullary leukemia lasting 8 months. After systemic disease relapse, he received autologous humanized CAR22-41BB-CD3ζ-tEGFR-T cells and achieved complete remission (CR) with incomplete blood count recovery (CRi) with minimal residual disease (MRD) negativity in the bone marrow and shrinkage of extramedullary leukemia. Over 2 months later, he experienced a relapse of the systemic disease and he received autologous murine CAR19-41BB-CD3ζ-mIL15-T cells and achieved CRi_MRD-_ lasting 5 months with the strongest expansion and persistence of CAR. Finally, on relapse of CD19^−^ medullary disease, he received allogeneic humanized CAR22-41BB-CD3ζ-tEGFR-T cells but only achieved a transient decrease in the number of blasts. No CAR-T-cell-related encephalopathy syndrome was observed, and all side effects were manageable.

**Conclusion:**

Our report hints the feasibility and safety of CD19 CAR-T cell expressing membrane-bound IL-15 for patient with B-ALL even if relapsed after multiple CAR-T-cell therapies.

## Introduction

Chimeric antigen receptor (CAR)-T cells have been remarkably successful in treating B-cell acute lymphoblastic leukemia (B-ALL) (1–4). The high 12-month relapse rate is the major cause of treatment failure, with an early relapse of antigen-positive disease and subsequent relapses associated with antigen loss or decrease of antigen density (5, 6). Although reinfusions of CAR-T cells seem a reasonable approach to conquer antigen-positive relapse with loss of CAR-T cell persistence, this strategy has exhibited limited efficacy (7–9). Relapse of leukemia can be categorized into medullary and extramedullary (EM) relapse depending on the site of leukemia recurrence, with the latter accounting for approximately 30% of ALL cases after allo-HSCT (10). In previous trials, patients with EM involvement were frequently excluded from the study population (7, 11). Several small studies have demonstrated that CAR-T-cell traffic to sites of EM disease and exhibit clinical efficacy (12–16) although further investigation is needed.

IL-15 promotes CD8^+^ T and natural killer (NK) cell activation, proliferation, cytotoxicity, and survival—enhancing both specific and nonspecific antitumor activity (17). High serum IL-15 levels were associated with the effectiveness of CD19 CAR-T-cell treatment (18). Furthermore, transgenic expression of IL-15 in CAR-T cells exhibited improved proliferative capacity, persistence, and cytokine production in a preclinical study of glioblastoma (19). To our knowledge, no clinical study on CAR-T with mIL-15 expression has been reported. Herein, we report a case of B-ALL experiencing EM relapse after allo-HSCT with resistance to multiple chemotherapy and radiotherapy regimens. The patient totally received four times of single-dose intravenous infusions of CAR-T cells under disease progression, which were CAR19-CD28-CD3ζ, CAR22-41BB-CD3ζ-tEGFR, CAR19-41BB-CD3ζ-mIL15, and CAR22-41BB-CD3ζ-tEGFR-T cells, respectively.

### Case Report

**Figure 1:**
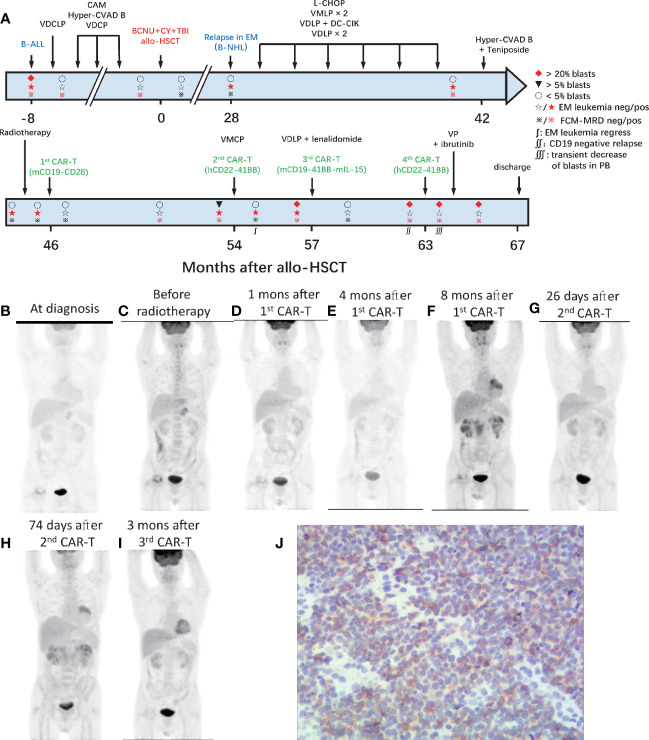
Process of treatment with CAR-T cells and clinical response. **(A)** The process of CAR-T-cell treatment and clinical response in the BM. The results of PET-CT are shown in **(B–I)**. **(B)** At diagnosis, multiple high metabolic lesions were detected in bilateral neck, axilla, mediastinum, abdominal, and retroperitoneal cavity. **(C)** Before radiotherapy, multiple lesions in the mediastinum and abdominal cavity were involved by EM leukemia, as well as the gastric wall. **(D)** One month after the 1st CAR-T-cell infusion, extramedullary leukemia achieved full resolution. **(E)** Four months after the 1st CAR-T infusion, extramedullary leukemia remained at full resolution. **(F)** Eight months after the 1st CAR-T infusion, the EM leukemia recurred in multiple lesions, including lymph nodes in the mediastinum and right inner mammary region, intrapericardial, bilateral kidney, retroperitoneal, perirenal, and pelvic peritoneum. **(G)** Twenty-six days after the 2nd CAR-T cell infusion, EM leukemia regressed. **(H)** Seventy-four days after the 2nd CAR-T cell infusion and 10 days before the 3rd CAR-T infusion, EM leukemia progressed. **(I)** Three months after the 3rd CAR-T cell infusion, EM leukemia achieved full resolution again. BM, bone marrow; EM, extramedullary; CAR, chimeric antigen receptor. **(J)** Immunohistochemical results of renal tissue showed weak positive CD22 in tumor cells.

A 39-year-old male patient complained of chest pain and abdominal distension for 1 month. He had been diagnosed with B-ALL 4 years prior. Leukemia blasts accounted for 94.1% in bone marrow (BM) and were immunophenotypically characterized as CD19^+^/CD34^+^/CD123^+^/c-IgM^+^/cCD79a^+^ and partially CD10^+^/CD20^dim^/HLA-DR^dim^/cTDT^+^. Positron emission tomography-computed tomography (PET-CT) revealed multiple high metabolic lesions in bilateral neck, axilla, mediastinum, abdominal, and retroperitoneal cavities. A biopsy of the right cervical lymph node and the immunohistochemical phenotype profiles indicated diffused TdT, CD43, BCL-2, Pax-5, and CD10, scattered CD3, CD79a, CD45RO, MPO, and CD15, focalized CD20 and CD21, individual CD1a, and 60% Ki-67^+^ cells. The expression of Bcl-6 and Mum-1 was negative. He achieved complete remission (CR) after one cycle of standard vincristine, daunomycin, cyclophosphamide, l-asparaginase, prednisone (VDCLP) induction. The postremission treatment included one cycle of cyclophosphamide, cytarabine, and 6-mercaptopurine (CAM), one cycle of hyper-CVAD B (methotrexate, leucovorin, sodium bicarbonate, cytarabine), and one cycle of vincristine, daunomycin, cyclophosphamide, prednisone (VDCP). The patient maintained CR but never achieved minimal residual disease (MRD) negativity in the BM by flow cytometry prior to transplantation. He underwent an HLA-identical sibling donor allo-HSCT subsequent to bis-chloroethyltrosourea, cyclophosphamide, and total body irradiation as the preconditioning regimen followed by cyclosporine A and short-term methotrexate for prophylaxis of graft *versus* host disease. The treatment progress is shown in **Figure 1A**. The characteristics of each CAR-T-cell infusion are summarized in **Table 1**. He achieved a CR with MRD negativity (CR_MRD−_) 1 month after transplantation. Unfortunately, the patient underwent EM relapse in the mediastinum 2 years after allo-HSCT (**Figure 1B**). The blasts of mediastinal mass expressed B cell markers (PAX5 and CD19) and naive cell markers (CD34, CD10, and TdT) by immunohistochemistry. Next, he received one cycle of L-asparaginase, cyclophosphamide, hydroxydaunorubicin, oncovin, prednisone (L-CHOP), two cycles of vincristine, mitoxantrone, L-asparaginase, prednisone (VMLP), one cycle of vincristine, idarubicin, L-asparaginase, prednisone (VDLP) combined with dendritic cells and cytokine-induced killer cells (DC-CIK) treatment, two cycles of VDLP. The EM disease did not regress and MRD tested positive (0.02%). After one cycle of hyperCVAD B+Teniposide, and tested MRD negative, but the EM leukemia progressed. PET-CT revealed multiple lesions in the mediastinum and abdominal cavity involved EM leukemia, with further evidence of gastric wall involvement (**Figure 1C**).

**Table 1:** Characterization of the four infusions of CAR-T cells.

	1st	2nd	3rd	4th
Clinical trial number	NCT02186860	NCT03262298	Compassionate use	NCT03262298
Vector^c^	Retroviral	Retroviral	Retroviral	Retroviral
CAR structure^c^	Anti-CD19 scFv (FMC63)-CD8-CD28-CD3ζ	Anti-CD22 scFv (m971)-CD8-4-1BB-CD3ζ-tEGFR	Anti-CD19 scFv (FMC63)-CD8-4-1BB-CD3ζ-mIL15	Anti-CD22 scFv (m971)-CD8-4-1BB-CD3ζ-tEGFR
scFv	Murine	Humanized	Murine	Humanized
Derived	Autologous	Autologous	Autologous	Allogenic
Classification	CD3^+^ 98.8%	CD3^+^ 98.5%	CD3^+^ 99.8%	CD3^+^ 99.1%
	CD8^+^ 16.7%	CD8^+^ 37.5%	CD8^+^ 76.4%	CD8^+^ 34.2%
	CD4^+^ 66.9%	CD4^+^ 57.9%	CD4^+^ 20.8%	CD4^+^ 57.5%
CD4/CD8	4.01	1.54	0.27	1.68
Memory T cells^b^	21.49%	35.28%	35.6%	39.83%
Total cells dose	3.79 × 10^7^	1.24 × 10^8^	5.25 × 10^8^	1.576 × 10^8^
Efficiency	66.4%	50.1%	12.4%	79.1%
Total CAR^+^ cells dose	2.49 × 10^7^	6.12 × 10^7^	6.50 × 10^7^	1.24 × 10^8^
CAR^+^ cells (kg)	0.5 × 10^6^	1 × 10^6^	1 × 10^6^	2 × 10^6^
Pretreatment	–	VMCP	Lenalidomide + VDLP	–
Blasts% pre-CAR-T cell infusion	EM^+^ and MRD^−^	EM^+^ and 5.5% in BM	EM^+^ and 96% in BM	EM free; 96.5% in BM
ECOG score pre-CAR-T cell infusion	1	1	2	2
Immunotype pre-CAR-T-cell infusion	Positive for CD19, CD38, CD34, TdT, CD81dim, CD9, CD22, negative for CD10, CD13, CD33, CD20, CD138	Positive for CD19, cCD79a, CD38, TdT, CD22, partial positive for CD34, CD10, negative for CD20	Positive for CD38, cCD79a, TDT, CD81, CD22, partial positive for CD10, CD34	Positive for HLA-DR, CD34, cCD79a, CD38, cTdT dim, CD81, CD22, CD24, partial positive for CD10, negative for CD19, CD20
Maximum of CAR copy (copies/µg gDNA)	2.64 × 10^3^	4.95 × 10^4^	5.81 × 10^5^	2.53 × 10^3^
Precondition	CyFlu^a^	CyFlu	CyFlu	CyFlu
Response in BM	MRD^−^ CR (8 months)	MRD^−^ CR (1 months)	MRD^−^ CRi (5 months)	Transient blasts decrease in PB
Response in extramedullary	Full resolution	Regress	Full resolution	–
Expression of CD19/CD22 before treatment	CD19^+^CD22^+^	CD19^+^CD22^+^	CD19^+^CD22^+^	CD19^−^CD22^+^
Reasons of relapse	The loss of CAR	The loss of CAR	CD19 antigen escape	The loss of CAR
CAR detected when relapse	No	No	Yes	No
CRS	Yes	Yes	Yes	Yes
Severe CRS	No	No	No	Yes
Glucocorticoid	No	No	No	Methylprednisolone

CAR, chimeric antigen receptor; scFv, single-chain fragment variable; tEGFR, truncated human epidermal growth factor receptor (EGFR) polypeptide; MRD, minimal residual disease; VMCP, vincristine, melphalan, cyclophosphamide, prednisone; VDLP, vincristine, melphalan, cyclophosphamide, prednisone; CyFlu, cyclophosphamide, fludarabine; CR, complete remission; CRS, cytokine release syndrome; EM, extramedullary.

a Cy, cyclophosphamide; Flu, fludarabine; Cy, 1,000 mg/m^2^/day, d1; Flu, 30 mg/m^2^/day, d1-3; dose of the 1st Flu in d2 is 80 mg, the others are 50 mg.

b Immunophenotyping of memory T cells is CD45RA^+^CD62L^+^ and CD45RA^−^CD62L^+^.

c See the Materials and Methods section in the **Supplementary Material**.

After providing informed consent, he was enrolled in a clinical trial (NCT02186860) and was admitted for CAR-T-cell treatment. He underwent leukapheresis in the preparation of CAR-T-cell infusions. The excess cells were cryopreserved and used for the next two CAR-T-cell preparation. He received four cycles of intra-abdominal lymph node intensity-modulated radiotherapy and EM disease remained in the lymph nodes in left lower pulmonary aorta, bilateral axillary, and mediastinal on contrast-enhanced CT. One month later, he received FC (cyclophosphamide, at day −4; fludarabine, at days −4 to −2) preconditioning and autologous murine CAR19-CD28-CD3ζ-T-cell infusion administered (day 0). EM leukemia full resolution was confirmed 1 month later by PET/CT (**Figure 1D**). However, the MRD positivity in the BM increased to 0.02% at 4 months with EM leukemia free (**Figure 1E**) followed by blasts in the BM and MRD increased to 5.5% (CD19^+^CD22^+^) and 6.08% at 8 months after CAR-T-cell infusion. PET/CT revealed the EM leukemia recurred in multiple lesions, including lymph nodes in the mediastinum and right inner mammary region, intrapericardial, bilateral kidney, retroperitoneal, perirenal, and pelvic peritoneum (**Figure 1F**). Renal biopsy revealed the immunophenotype of leukemia cells was CD19^+^, with scattered CD20^+^/CD22^+^/CD34^+^/PAX-8^+^/CD79a^+^/TdT^+^/CD10^+^/CD3^−^/CD33^−^/CD99^−^, and CD1a^−^ (**Figure 1J**; **Supplementary Figure S1**).

After one cycle of vincristine, melphalan, cyclophosphamide, prednisone (VMCP) to reduce tumor burden, the patient received FC preconditioning and autologous humanized CAR22-41BB-CD3ζ-tEGFR-T-cell infusion (NCT03262298). He achieved CR_MRD−_ in the BM with transfusion-independent and EM leukemia burden marked decreased on PET/CT 1 month later (**Figure 1G**). Nonetheless, CD19^+^CD22^+^ blasts quickly rose to 96% in the BM along and EM leukemia recurred more than 1 month later (**Figure 1H**).

**Figure 2:**
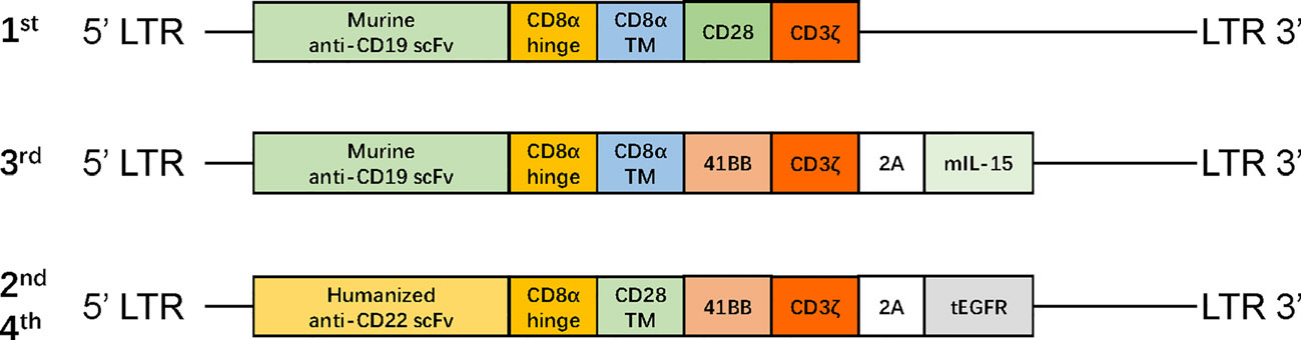
The structure of retroviral vectors encoding CARs. The CAR19-CD28-CD3ζ consisted of murine anti-CD19 scFv, a CD8 hinge region, CD28 transmembrane and cytoplasmic domain, and a CD3ζ cytoplasmic region. The CAR19-41BB-CD3ζ-mIL15 consisted of humanized anti-CD19 scFv, a CD8 hinge and transmembrane region, 4-1BB costimulatory domain, and a CD3ζ cytoplasmic region; mIL-15 is connected with CAR gene *via* a P2A peptide. The CAR22-41BB-CD3ζ-tEGFR consisted of humanized anti-CD22 scFv, a CD8 hinge and transmembrane region, 4-1BB costimulatory domain, and CD3ζ cytoplasmic region; tEGFR is integrated with CAR gene through a P2A peptide. mIL-15, membrane IL-15; EGFR, human epidermal growth factor receptor; tEGFR, truncated EGFR polypeptide; scFv, single-chain fragment variable.

After one cycle of VDLP and lenalidomide to reduce tumor burden, the patient received FC preconditioning and the autologous murine CAR19-41BB-CD3ζ-mIL15-T-cell infusion for compassionate use. He achieved transfusion-independent CRi_MRD−_ in the BM, which was 1 month later and achieved full resolution of EM leukemia 3 months later on PET/CT (**Figure 1I**). Five months after CAR-T-cell infusion, the blasts count rose to 86.38% in the BM and were CD19^−^CD22^+^. No evidence of EM leukemia was found on computed tomography (CT) or ultrasound examination. CARs design is shown in **Figure 2**.

At relapse, the patient is in a state of incomplete hematopoietic recovery and the cryopreserved cells are depleted. Failing to meet the conditions for preparation of autologous CAR-T cells, he received predonor-derived humanized CAR22-41BB-CD3ζ-tEGFR-T-cell infusion (NCT03262298) after FC preconditioning and achieved a transient decrease in blasts in the peripheral blood (PB). Unfortunately, due to loss of CAR following methylprednisolone, the disease progressed within 28 days. The patient then received one cycle of vincristine, prednisone (VP) as reinduction chemotherapy with ibrutinib. After more than 3 months, the patient did not achieve remission. He abandoned treatment and was discharged for financial reasons.

### Toxicities, Persistence of CAR-T Cells, and Tumor Antigen Expression

**Figure 3:**
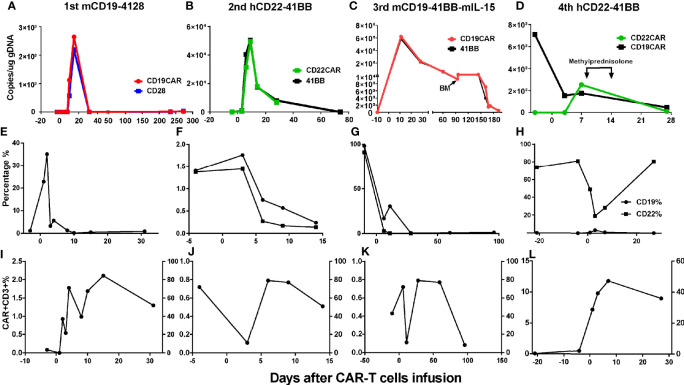
Changes of CAR% and blasts% in PB after CAR-T cell infusions. **(A, E, I)** After the 1st infusion, the copies of CAR reached the expansion peak on d15 and fell to undetectable on d31 and remained almost undetectable during relapse 8 months later. The expansion of CAR copies was coincided with the drop of CD19^+^ blasts in PB. **(B, F, J)** After 2nd infusion, the expansion peak was within 14 days and coincided with the drop of CD19^+^CD22^+^ blasts in PB. After about 2 months, the CAR copies gradually fell to being undetectable. **(C, G, K)** After the 3rd infusion, the copies of CAR ascended to expansion peak about 10 days later in keep with the drop of CD19^+^CD22^+^ blasts in PB and could be detected after CD19^−^ relapse lasting more than 5 months. **(D, H, L)** The nadir of CD22^+^ blasts in PB were corresponding with the peak of CAR expansion. The drop of CART22 was in keeping with CART19, eliminated by methylprednisolone (40 mg d1-3, 20 mg d 4-6).

**Figure 4:**
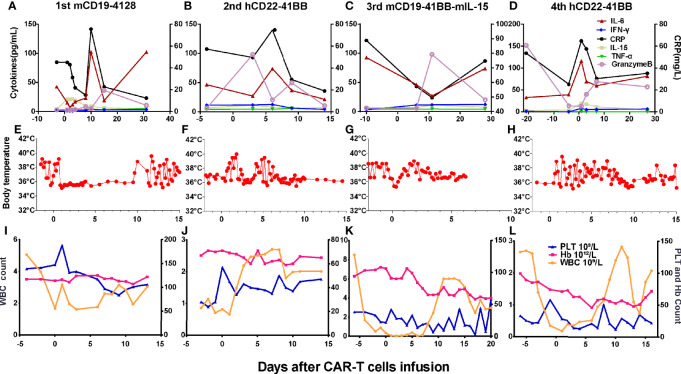
Changes of cytokines, CRP, body temperatures, and hemograms after CAR-T cell infusions. **(A, E)** After the 1st infusion, the patient developed fever, elevation of IL-6, granzyme B, and CRP on day 9. **(B, F)** After the 2nd infusion, the patient developed fever, chill on the day 1. The level of IL-6, granzyme B, and CRP elevated on days 3 and 6, respectively, and then dropped to baseline on day 14. **(C, G)** After the 3rd infusion, the patient developed fever, chill quickly, then elevation of IL-6, granzyme B, and CRP successively 1 week later. **(D, H)** The last infusion caused fever, elevation of granzyme B, and CRP on day 1 and IL-6 thereafter. **(I–L)** Changes of hemogram after the CAR-T-cell infusions.

Grades (G) of treatment-emergent adverse events (AEs) after each infusion are shown in **Supplementary Table S1**. No >G2 AE were observed after the first and second infusions. After the first infusion of CAR-T cells, the patient developed G1 cytokine-release syndrome (CRS) (**Figures 4A, E**). The copies of CAR reached the expansion peak at day 14 and fell to undetectable levels within 1 month (**Figure 3A**). After the second infusion of CAR-T cells, the patient developed G1 CRS as well, which improved after symptomatic treatment (**Figures 4B, F**). The CAR copies peaked within 14 days and gradually became undetectable after about 2 months (**Figure 3B**). After the third infusion of CAR-T cells, the patient was evaluated as G2 CRS on day 6 and was cured by anti-infection and symptomatic supportive treatment (**Figures 4C, G**). The copies of CD19 CAR reached an expansion peak approximately 10 days later and were detected even after CD19^−^ relapse (**Figure 3C**). The final infusion of CAR-T cells caused fever, and rapid elevation of granzyme B, CRP, and IL-6, evaluated as G4 CRS on day 10 (**Figures 4D, H**). He underwent treatment with methylprednisolone, which caused rapid loss of CAR copies (**Figure 3D**). No nervous system disorder was observed during any infusion. After each infusion of CAR-T cells, the expansion of CAR copies coincided with the drop in PB blasts (**Figures 3E–L**). After each relapse, CD22 site density remained almost unchanged (**Supplementary Figure S2**). Hemogram changes during treatments are shown in **Figures 4I–L**.

## Discussion and Conclusions

Herein, we report a refractory B-ALL patient with EM relapse after allo-HSCT who received repeat CAR-T-cell therapies. Both CAR19-CD28-CD3ζ-T and CAR22-41BB-CD3ζ-tEGFR-T cell only achieved short persistence following by CD19^+^ relapse. By contrast, after systemic relapse, CAR19-41BB-CD3ζ-mIL15-T cell achieved CRi_MRD−_ and full resolution of EM leukemia lasting 5 months with transfusion independence, followed by high CAR persistence and CD19^−^ relapse.

The results of clinical trials indicate that CAR containing CD28 persists shorter than CAR with 4-1BB (20, 21). Preclinical and clinical data confirm that CAR-T cells with 4-1BB tend to expand in patients at later time points compared with those with CD28 (22–24). The short persistence of the first CAR-T-cell infusion was possibly associated with CD28 costimulatory domain. CAR copies fell to undetectable levels 1 month after infusion on achieving CR_MRD−_. Although minimal CAR expansion and persistence after the first infusion was maintained, CAR19-CD28-CD3ζ-T cells exerted a sustained control of leukemia, possibly related to prior radiotherapy. In preclinical studies, radiotherapy enhanced the efficacy of CAR-T cells by promoting their migration to the tumor site and increased effector functions in glioblastoma models (25) and by enhancing the sensitivity of CAR-T cell to antigen-negative tumor cells to reduce immune escape (26). Radiotherapy as a bridging strategy for CART19 in high-risk lymphoma has been proven to be clinically safe (27). Our report shows that radiotherapy bridging to CAR-T cells is feasible, but whether radiotherapy enhances the infiltration and activity of CAR-T cells in extramedullary lesions needs further investigation.

Recent studies have indicated that humanized CART19 could induce remission in patients with relapsed/refractory B-ALL, especially in patients who received a reinfusion of murine CAR-T cells (28). Thus, considering the loss of CAR possibly caused by murine CAR, humanized CAR22-41BB-CD3ζ-tEGFR-T cells have also been used. CD22 CAR-T cells exhibited high response rates in B-ALL patients after failure of CD19 CAR-T cells (29). However, the patient progressed rapidly after first CART22 infusion with loss of CAR without achieving full resolution of EM leukemia. Diminished CD22 site density is sufficient to permit escape of leukemia from CD22-directed CAR therapy rather than total loss of surface expression of CD19 (29) but did not diminish on relapse following CART22 infusions in our study. CD19 CAR-T-cell efficacy of CD22 CAR-T cells may be related to the low level of CD22 expression in EM sites and the affinity of the single-chain fragment variable, which needs further investigation.

For the first time, we used CD19 CAR-T cells with transgenic expression of membrane IL-15 clinically. Although the tumor burden before infusion was extremely high, the patient achieved CRi_MRD−_ with the highest expansion and longest persistence of CAR copies and revisable toxicity after CAR19-41BB-CD3ζ-mIL15-T-cell infusion. Moreover, remission induced by subsequently murine CART19 demonstrated that endogenous factors or immunosuppression but not the production of human antimouse antibodies resulted in relapse after the first murine CART19 infusion. The serum level of IL-15 was detected after each infusion of CAR-T and maintained at low level without significant change. Since CAR-T cells express mIL-15, it is understandable that the serum level of IL-15 has not changed. It is our limitation that mIL-15 expression on CAR-T cells was not detected in our clinical study. However, transgenic expression of IL-15 in CAR-T cells exhibited improved proliferative capacity and persistence preclinically (19). Thus, continuous expansion of CAR-T cells may indirectly reflects the function of IL-15 in our study. Furthermore, excessive expansion of CAR19-41BB-CD3ζ-mIL15-T cells possibly cause sustained inflammation and cytokine production, which could explain the incomplete hematopoietic recovery. The patient underwent CD19-negative relapse with CAR-T-cell persistence, illustrating CAR19-41BB-CD3ζ-mIL15-T cells possibly exerted a stronger immunopressure. Although antigen-negative relapses are not uncommon in B-ALL with CAR-T therapy, previous report has also highlighted the presence of antigen loss variants following infusions of anti-IL13Rα2 CAR-T cells with IL-15 expression, although the mechanisms remain unknown (19). Coexpression of mIL-15 enhanced effector functions, engraftment, and tumor control of CAR-T cells and remodeled the tumor microenvironment (TME) to favor tumor control, including NK cell activation and reduced presence of M2 macrophages (30). In our case, after treatment with CD19 CAR-T cells with mIL-15 expression, the patient relapsed without EM leukemia, which may relate to TME reprogramming, and thus requires further investigation.

The final infusion of CD22 CAR-T cell achieved inferior leukemia responses and expansion of CAR. High tumor burden in the BM, high dosage of CAR-T cells, and thrombocytopenia before lymphodepletion might contribute to the development of severe CRS after the fourth infusion of CAR-T cells (31). Drugs including alemtuzumab and corticosteroids may abrogate CAR-T cell expansion and persistence (32, 33). Thus, treatment with glucocorticoids caused the rapid reduction of CAR-T cells.

This study provides a rare perspective that compares the outcomes of different CAR-T cells in the same patient. Our report hints that it is feasible and safe to infuse CD19 CAR-T-cell-expressing membrane-bound IL-15 for patient with B-ALL even if relapsed after multiple CAR-T-cell therapies.

## Materials and Methods

See the *Materials and Methods* section in the **Supplementary Material**.

## Data Availability Statement

The original contributions presented in the study are included in the article/**Supplementary Material**. Further inquiries can be directed to the corresponding authors.

## Ethics Statement

The studies involving human participants were reviewed and approved by the Institutional Review Board at the Fifth Medical Center of Chinese PLA General Hospital. The patients/participants provided their written informed consent to participate in this study.

## Author Contributions

LH designed the trial and experiments and revised the manuscript. YaS performed experiments, analyzed the data, and wrote the paper. YoS, HN, and YW managed the patients. NL, YL, JC, JN, JH, ZQ, and BZ analyzed the data. All authors contributed to the article and approved the submitted version.

## Funding

This work was supported by grants from the Health Care Project of Army (19BJZ37).

## Conflict of Interest

The authors declare that the research was conducted in the absence of any commercial or financial relationships that could be construed as a potential conflict of interest.

## Publisher’s Note

All claims expressed in this article are solely those of the authors and do not necessarily represent those of their affiliated organizations, or those of the publisher, the editors and the reviewers. Any product that may be evaluated in this article, or claim that may be made by its manufacturer, is not guaranteed or endorsed by the publisher.

## Supplementary Material

Supplementary Figure 1 Immunohistochemical results of renal tissue showed positive CD19, CD34, and TdT in tumor cells.

Supplementary Figure 2 CD22 site density when relapse after CAR T cells.
