# Supplementary figures and images for: CD19 CAR-T Cells With Membrane-Bound IL-15 for B-Cell Acute Lymphoblastic Leukemia After Failure of CD19 and CD22 CAR-T Cells: Case Report
Source: Front Immunol. 2021 Oct 7;12:728962. doi: 10.3389/fimmu.2021.728962 (PMC8530183; doi:10.3389/fimmu.2021.728962)

CD19

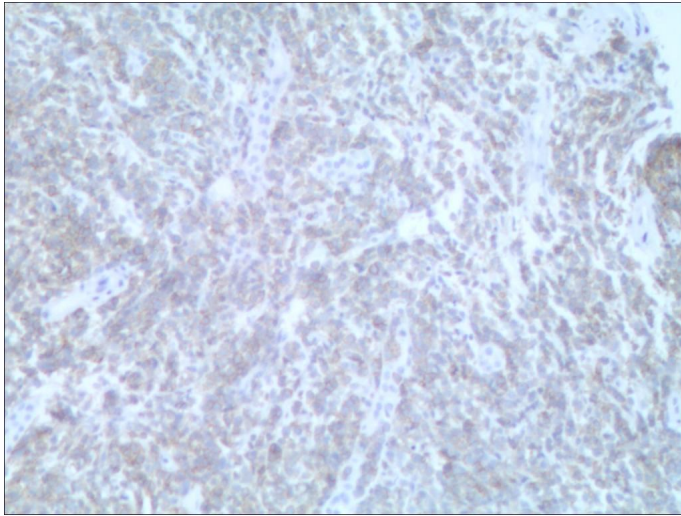

TdT

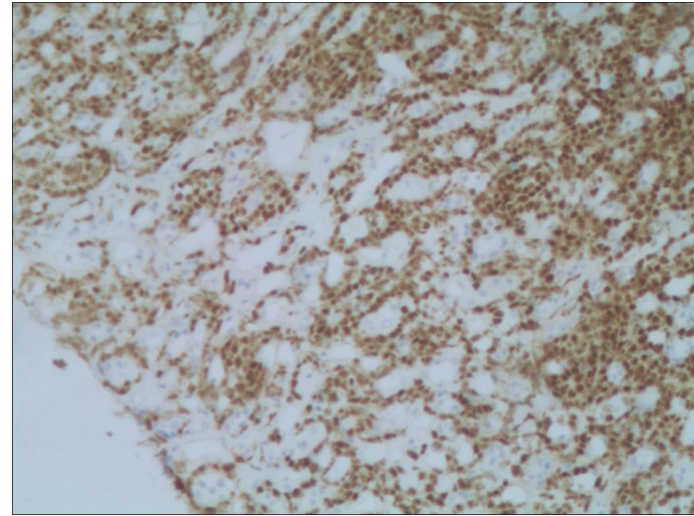

CD34

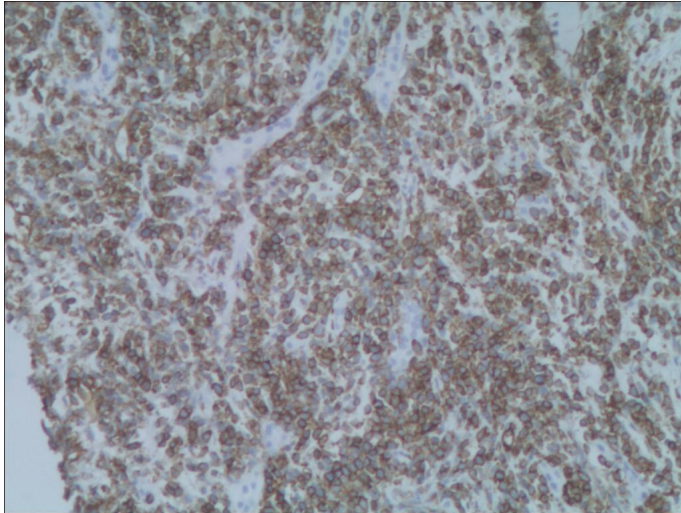

Supplement: Supplementary Figure 1 — Immunohistochemical results of renal tissue showed positive CD19, CD34, and TdT in tumor cells. [file Image_1.pdf]

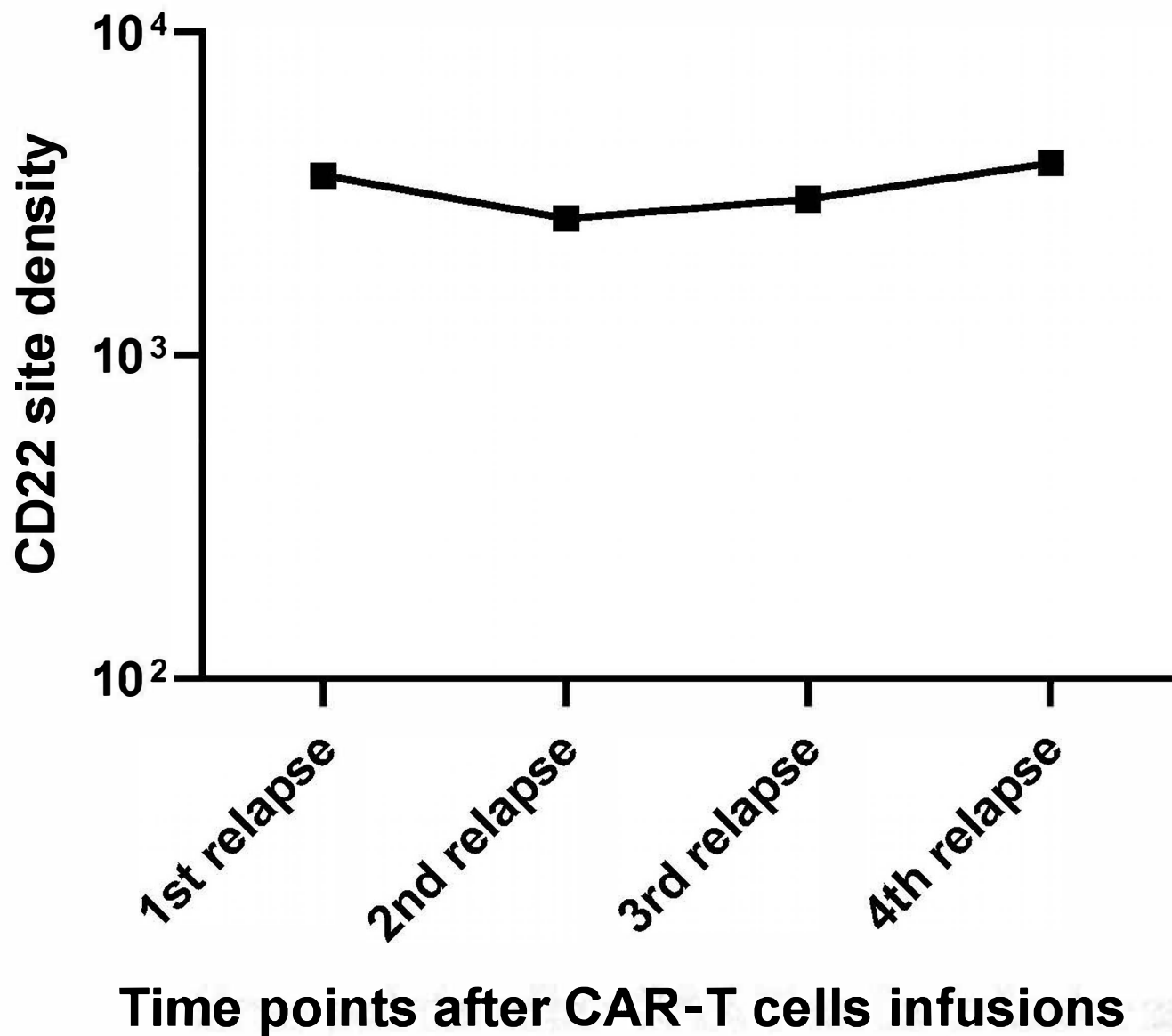

Supplement: Supplementary Figure 2 — CD22 site density when relapse after CAR T cells. [file Image_2.pdf]
