# Supplementary material for: CD19 CAR-T Cells With Membrane-Bound IL-15 for B-Cell Acute Lymphoblastic Leukemia After Failure of CD19 and CD22 CAR-T Cells: Case Report
Source: Front Immunol. 2021 Oct 7;12:728962. doi: 10.3389/fimmu.2021.728962 (PMC8530183; doi:10.3389/fimmu.2021.728962)
Supplement: Supplementary file 3 [file DataSheet_1.docx]

**Materials and Methods**

***Generation of retroviral vectors***

The retroviral vectors encoding anti-CD19 or CD22 CARs were constructed based on a modified Moloney Murine Leukemia Virus (Mo-MLV) vector described previously [1]. The CAR19-CD28-CD3ζ consisted of murine anti-CD19 single chain fragment variable (scFv), CD28 extracellular, transmembrane, and cytoplasmic domain, and CD3ζ cytoplasmic region [2]; The CAR22-41BB-CD3ζ-tEGFR consisted of humanized anti-CD22 scFv, CD8 hinge region, 4-1BB costimulatory domain, and CD3ζ cytoplasmic region; Truncated human Epidermal Growth Factor Receptor (EGFR) polypeptide (tEGFR) was integrated with CAR gene through a P2A peptide. The creation of the anti-CD22 scFv (m971) was previously described [3]. The production of clinical-grade retroviral was described previously [4]. The CAR19-41BB-CD3ζ-mIL15 consisted of humanized anti-CD19 scFv, CD8 hinge region, 4-1BB costimulatory domain, and CD3ζ cytoplasmic region [5]; membrane IL-15 (mIL-15) through a P2A peptide .

***CAR T cell production***

Thawed PBMC from the eligible donor were cultured in T cell medium (TCM) containing X-vivo15 serum-free medium (Lonza, Allendale NJ), 5% (vol/vol) GemCell human serum antibody AB (Gemini Bio Products, West Sacramento CA), 1% (vol/vol) Glutamax-100× (Gibco Life Technologies), 10mM HEPES buffer (Corning), and 2%（m/V）N-Acetyl-L-cysteine (Sigma). The culture was supplemented with 50-100 IU/mL human IL-2. The PBMC were activated, expanded, transducted, and cultured as previously described [6]. At days 8-12, the CAR T cell was ready for the production release testing: CAR+ >30% and expansion at least 5-fold under the stimulation of CD3/28 beads stimulation in vitro. Then, we could prepare for infusion or thawing at the appropriate time.

***Biomarker analysis***

Multi-parametric flow cytometry was used for analysis of various PBMC and CAR-T samples. The CAR-T were stained with fluorescent-labeled antibodies against CD3, CD4, CD8, CD45RA, and CD62L (BioLegend, San Diego, CA). single chain antibody fragment (scFv) of CAR19 was detected with anti-CAR19 scFv antibodies (BioLegend, San Diego, CA) to directly represent the detection of CAR19. tEGFR was detected with anti-EGFR antibodies (BioLegend, San Diego, CA) to indirectly represent the detection of CAR22.

The presence, expansion, and persistence of CARs in the blood were monitored by quantitative PCR. Genomic DNA was isolated from PBMC samples using MiniBEST Universal Genomic DNA Extraction Kit (Takara), quantified by a spectrophotometer, and stored at -80°C. The qPCR analysis on genomic DNA samples was performed to detect the integrated CAR transgene sequence.

1. B. Engels, H. Cam, T. Schüler, S. Indraccolo, M. Gladow, C. Baum, T. Blankenstein, and W. Uckert, Retroviral vectors for high-level transgene expression in T lymphocytes. *Human Gene Therapy* (2003).14:1155.
2. Kalos M., Levine B.L., Porter D.L., Katz S., Grupp S.A., Bagg A., June C.H. T cells with chimeric antigen receptors have potent antitumor effects and can establish memory in patients with advanced leukemia. *Sci Transl Med* (2011).3:95ra73.
3. Xiao X, Ho M, Zhu Z, Pastan I, Dimitrov DS. Identification and characterization of fully human anti-CD22 monoclonal antibodies. *mAbs*. (2009).1(3):297–303.
4. Lee D.W., Gardner R., Porter D.L., Louis C.U., Ahmed N., Jensen M., Grupp S.A., Mackall C.L. Current concepts in the diagnosis and management of cytokien release syndrome. *Blood* (2014).142:188–195.

[5] Z. Cheng, R. Wei, Q. Ma, L. Shi, F. He, Z. Shi, T. Jin, R. Xie, B. Wei, J. Chen, H. Fang, X. Han, J.A. Rohrs, P. Bryson, Y. Liu, Q.J. Li, B. Zhu, and P. Wang, In Vivo Expansion and Antitumor Activity of Coinfused CD28-and 4-1BB-Engineered CAR-T Cells in Patients with B Cell Leukemia. *Mol Ther* (2018). 26(4): 976–985.

[6] Y Sun, J Chen, Y Liu, B Li, Q Wang, H Fang, L Zhang, H Ning, P Wang, H Chen, L Hu and B Zhang, Donor-Derived CD123-Targeted CAR T Cell Serves as a RIC Regimen for Haploidentical Transplantation in a Patient With FUS-ERG+ AML. *Front. Oncol* (2019). 9:1358.
