# Supplementary material for: CD19 CAR-T Cells With Membrane-Bound IL-15 for B-Cell Acute Lymphoblastic Leukemia After Failure of CD19 and CD22 CAR-T Cells: Case Report
Source: Front Immunol. 2021 Oct 7;12:728962. doi: 10.3389/fimmu.2021.728962 (PMC8530183; doi:10.3389/fimmu.2021.728962)
Supplement: Supplementary file 4 [file Table_1.docx]

**Supplementary Table Grades of treatment-emergent adverse events after each infusion.**

| **Time of Infusion** | **1^st^** | **2^nd^** | **3^rd^** | **4^th^** |
| --- | --- | --- | --- | --- |
| Fever | 1 | 1 | 1 | 1 |
| Chill | 1 | 1 | 1 |  |
| Cytokine release syndrome | 1 | 1 | 2 | 4 |
| Tracheitis | 2 |  |  |  |
| Skin infection |  | 2 |  |  |
| Hypotension |  |  | 3 |  |
| Pneumonia |  |  | 2 | 2 |
| Pancreatitis |  |  | 3 |  |
| Cholecystitis |  |  | 2 |  |
| Nausea |  |  | 1 |  |
| Headache | 1 |  |  |  |
| Enchphalopathy |  |  |  |  |
| Nerous system disorder |  |  |  |  |
| Hydropericardium |  |  |  | 2 |
| Hydrothorax |  |  |  | 2 |
| Atelectasis |  |  |  | 1 |
| Flu like symptoms |  |  |  | 1 |

Adverse event is graded per CTCAE, version 4.03; Cytokine release syndrome was graded per a modified grading system described by Lee et al.
